# Circulating miR-16 as an Early Biomarker of Subclinical Myocardial Strain Impairment in Pediatric Primary Hypertension

**DOI:** 10.3390/ijms27062806

**Published:** 2026-03-20

**Authors:** Michał Szyszka, Radosław Pietrzak, Klaudia Obsznajczyk, Karolina Skubisz, Ceren Eyileten, Piotr Skrzypczyk

**Affiliations:** 1 Department of Pediatrics and Nephrology, Doctoral School, Medical University of Warsaw, 02-091 Warsaw, Poland; michal.szyszka@wum.edu.pl; 2 Department of Pediatric Cardiology and General Pediatrics, Medical University of Warsaw, 02-091 Warsaw, Poland; radoslaw.pietrzak@wum.edu.pl (R.P.);; 3 Department of Laboratory Diagnostics and Clinical Immunology of Developmental Age, Pediatric Hospital of Medical University of Warsaw; 02-091 Warsaw, Poland; 4 Laboratory of Genetics, University Center for Laboratory Medicine, University Clinical Centre of the Medical University of Warsaw, 02-091 Warsaw, Poland; 5 Department of Experimental and Clinical Pharmacology, Center for Preclinical Research and Technology CEPT, Medical University of Warsaw, 02-091 Warsaw, Poland; 6 Genomics Core Facility, Centre of New Technologies, University of Warsaw, 02-097 Warsaw, Poland; 7 Department of Pediatrics and Nephrology, Medical University of Warsaw; 02-091 Warsaw, Poland

**Keywords:** microRNA, miR-16-5p, miR-27b-3p, global longitudinal strain, primary hypertension, children, adolescents, cardiac alterations

## Abstract

The role of circulating microRNAs in the pathophysiology of cardiac remodeling in primary hypertension (PH) remains incompletely understood. Left ventricular global longitudinal strain (LV GLS) is a sensitive marker of subclinical systolic dysfunction and can be used to monitor early cardiac involvement in cardiovascular and renal diseases. To the best of our knowledge, this is the first study to demonstrate an association between circulating miR-16 and LV GLS in children. The study aimed to evaluate the expression levels of miR-16-5p, -21-5p, -27a-3p, -27b-3p, -133a-3p, and -145-5p in untreated children with PH and examine their associations with LV GLS. 50 children with PH and 57 normotensive controls were evaluated for circulating microRNA expression levels and echocardiographic parameters, including LV GLS. Comprehensive anthropometric, biochemical, blood pressure, and arterial indices were also assessed. Among the analyzed microRNAs, miR-16-5p exhibited a positive association with LV GLS (R = 0.305, *p* = 0.031), whereas miR-27b-3p demonstrated a negative association (R = −0.330, *p* < 0.001). Compared with controls, hypertensive children exhibited significantly higher (i.e., less negative) LV GLS (r = 0.29, *p* = 0.002), indicating early systolic dysfunction occurring already at an early stage of the disease. In conclusion, these findings support the idea that specific microRNAs might play a differential role in early myocardial functional alterations in pediatric PH. Higher miR-16 expression levels may be associated with impaired myocardial deformation, potentially reflecting its involvement in early maladaptive myocardial remodeling. Furthermore, LV GLS may represent a sensitive and clinically informative marker of early myocardial dysfunction beyond traditional echocardiographic parameters in this population.

## 1. Introduction

MicroRNAs are small, non-coding RNAs that regulate gene expression and influence multiple, interconnected biological pathways [1,2]. Dysregulation of microRNAs has been implicated in cardiovascular remodeling and the development of related cardiovascular diseases [3]. Because they can be detected extracellularly, e.g., in plasma [4], circulating microRNAs have emerged as promising biomarkers of cardiovascular stress and early myocardial dysfunction [3].

Arterial hypertension (AH) in children is an increasing medical concern [5]. The prevalence of childhood AH increases during early adolescence, peaking around puberty (7.89% among teenagers aged 14 years) and subsequently declining in late adolescence (3.28% at 19 years) [5]. There is growing evidence that elevated blood pressure often begins in early life and continues into adulthood [6]. AH contributes to structural and functional changes in the heart, kidneys, and blood vessels [7]. Therefore, early identification and appropriate management are essential to prevent long-term cardiovascular complications. Unlike in adults, where hard endpoints such as strokes and myocardial infarctions are common, in children with primary hypertension, the presence of hypertension-mediated organ damage (HMOD) is the most important indicator for initiating pharmacological treatment [8]. Currently, left ventricular hypertrophy is considered the gold standard for assessing HMOD [9]. However, researchers are still seeking additional early markers of HMOD to improve prognosis and long-term treatment outcomes. One such marker may be global longitudinal strain (GLS) of the left ventricle (LV), as assessed by speckle tracking echocardiography (STE) [10].

MicroRNAs are increasingly recognized as key regulators and potential biomarkers of hypertensive heart disease [3,11]. Several circulating miRNAs have been associated with left ventricular hypertrophy (LVH) and subclinical myocardial remodeling in adults [12].

MiR-16-5p is a member of the miR-15 family. It is processed from the miR 15a/16-1 genomic cluster, which is located on human chromosome 13 [13]. Some studies have shown that it may be linked to mechanisms relevant to cardiovascular injury caused by hypertension, including the proliferation and migration of vascular smooth muscle cells related to angiotensin II [14]. Another study has shown that miR-16-3p can lead to the development of hypertensive heart disease by mediating sympathetic signaling in cardiomyocytes and vascular smooth muscle through the α1A-adrenergic receptor, which is encoded by the *ADRA1A* gene [15]. However, its exact role and whether it acts positively or negatively has not yet been fully elucidated.

MiR-27b-3p has been implicated in hypertensive cardiac remodeling. In adults, circulating miR-27b is elevated in patients with echocardiographic LVH and differentiates hypertensive individuals with and without LVH [16]. Experimental studies further demonstrate its involvement in pressure-overload-induced remodeling, although emerging data suggest that miR-27b may also exert context-dependent regulatory effects on endothelial function and myocardial adaptation [17].

Notably, miR-21 has been linked to interstitial fibrosis and, with miR-27a, to cardiac hypertrophy signaling [18,19]. MiR-133a was found to be involved in the regulation of cardiomyocyte hypertrophic responses, and miR-145 elicits a contribution to vascular smooth muscle remodeling [20,21].

**Figure 1:**
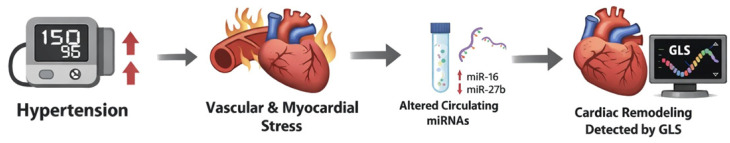
Conceptual model of hypertension-induced vascular stress, altered circulating microRNAs, and subclinical myocardial remodeling detected by LV GLS based on Sessen [22].

Thus, microRNAs have been implicated in key mechanisms of cardiovascular remodeling, including inflammation, fibrosis, endothelial dysfunction, and myocardial structural adaptation. In the setting of chronic pressure overload, alterations in circulating microRNA profiles may reflect early maladaptive or compensatory responses to vascular and myocardial stress [22]. Such molecular changes may influence structural abnormalities and be detectable through sensitive functional markers such as LV GLS (Figure 1) [22]. The relationship between circulating miR-16-5p and -27b-3p, and early myocardial dysfunction, particularly indices of myocardial deformation such as GLS, remains largely unexplored. Such data are virtually absent in children with PH. Given the increasing use of sensitive echocardiographic strain imaging, integrative studies combining circulating microRNAs with detailed myocardial phenotyping are urgently needed.

Thus, the aim of our study was to evaluate the expression levels of selected miRs (-16-5p, -21-5p, -27a-3p, -27b-3p, -133a-3p, and -145-5p) and the global longitudinal strain of the left ventricle in untreated pediatric patients with primary hypertension and to compare them with healthy peers.

## 2. Results

A total of 50 patients with primary hypertension and 57 healthy peers underwent complete microRNA evaluation and echocardiographic examinations and were included in the final analysis.

Table 1 summarizes the basic demographic, clinical, and biochemical parameters of both groups. Additional parameters and their comparison between the hypertensive and control groups, including *p*-values, are presented in Supplementary Table S1. The groups did not differ in terms of age or gender. However, significantly more patients in the study group had a family history of hypertension on their mother’s side. While patients did not differ in height, significant differences were found in body weight, BMI, waist and hip circumference, waist-to-hip ratio, ambulatory blood pressure measurement, and serum uric acid concentration. All participants had normal kidney function, and there were no differences between groups in terms of creatinine-based estimated glomerular filtration rate.

Table 2 and Supplementary Table S1 present a comparison between the study and control groups for measurements obtained during 24 h automated monitoring of arterial pressure, central blood pressure, and vascular parameters. The groups differed significantly in terms of 24 h measurements: systolic blood pressure (SBP), diastolic blood pressure (DBP), mean arterial pressure (MAP), pulse pressure (PP), central systolic blood pressure (cSBP), central diastolic blood pressure (cDBP), augmentation index corrected to 75 heartbeats (AIx@75HR), cardiac output (CO), total vascular resistance (TVR), reflection magnitude (RM), pulse wave velocity (PWV)—higher values in the group of patients with PH. No differences were found in 24 h heart rate (HR) and stroke volume (SV). The study group showed a significantly smaller nocturnal decrease in diastolic pressure, with no similar effect on systolic blood pressure. All ambulatory parameters of central pressure (AoSBP, AoDBP, AoMAP) were significantly higher in the group of patients with PH. In terms of vascular parameters, the study group had higher carotid–femoral PWV (cfPWV) and thicker carotid intima media thickness (cIMT); no differences between the groups were found in ambulatory augmentation index (Aix) measurements.

**Figure 2:**
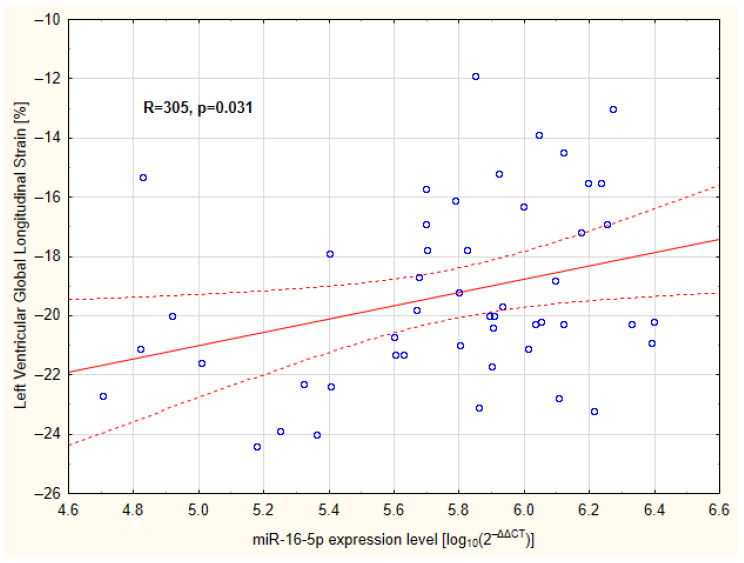
Relationship between global LV strain and relative miR-16-5p expression levels in children with primary hypertension. The figure shows the unadjusted Spearman correlation. The independent association was further confirmed in multivariable regression analysis (see Table 4). Blue circles represent individual study participants included in the analysis. Solid lines are linear regression lines, and dotted lines represent 95% confidence intervals.

Table 3 and Supplementary Table S1 show the comparison of echocardiographic parameters between the control and study groups. Patients with PH were characterized by higher left ventricular mass (LVM) and left ventricular mass index (LVMI), expressed as g/m^2.7^, as well as a higher LVMI Z-score. This group was also characterized by higher LV GLS values and significantly lower early diastolic mitral annular velocity (E’) wave of the septum and lateral wall. Additionally, the PH group had higher isovolumetric relaxation time (IVRT). The ejection fraction of all subjects was normal, and the study group did not differ significantly from the control group in this respect. The analysis restricted to the study group revealed a significant positive correlation between miR-16-5p and LV GLS (R = 0.305; *p* = 0.031; Figure 2). Patients with higher miR-16-5p levels showed correspondingly higher LV GLS values, suggesting a measurable association between this microRNA and myocardial deformation parameters in hypertensive pediatric patients.

**Figure 3:**
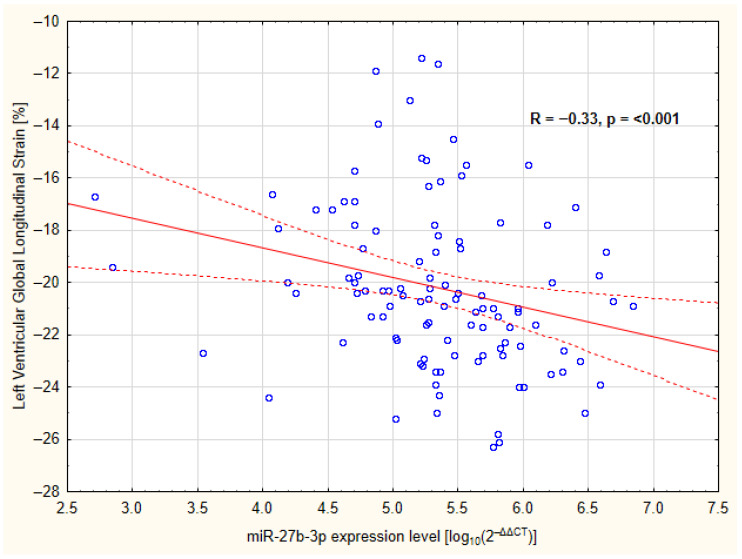
Relationship between global LV strain and relative miR-27b-3p expression levels in all children. The figure shows the unadjusted Spearman correlation. Blue circles represent individual study participants included in the analysis. Solid lines are linear regression lines and dotted lines represent 95% confidence intervals. LV—left ventricle.

In the overall cohort, circulating plasma miR-27b-3p levels were inversely associated with LV GLS values, suggesting that higher miR-27b-3p levels were associated with more favorable myocardial deformation indices (Figure 3).

No comparable correlations were identified for the other microRNAs assessed.

**Figure 4:**
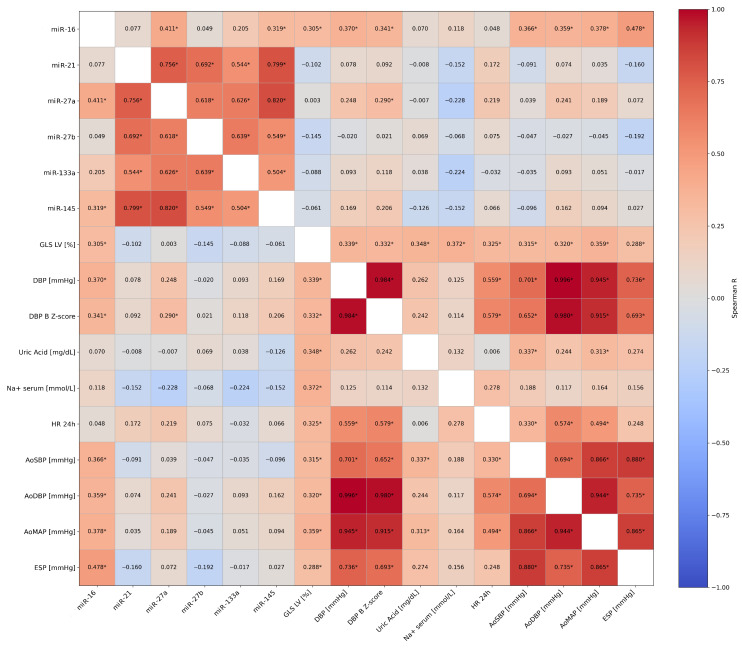
Unadjusted Spearman correlation matrix of the blood pressure, biochemical and echocardiographic parameters (including global left ventricle strain) and microRNA in the group of hypertensive children. Adjusted associations were subsequently evaluated using multivariable regression analysis. GLS LV—left ventricular global longitudinal strain; DBP—diastolic blood pressure; DBP Z-score—Z-score of diastolic blood pressure; HR 24 h—24 h mean heart rate; AoSBP—central (aortic) systolic blood pressure; AoDBP—central (aortic) diastolic blood pressure; AoMAP—central (aortic) mean arterial pressure; ESP—estimated end-systolic pressure. * indicates statistically significant correlations (*p* < 0.05).

Additionally, LV GLS exhibited significant positive correlations with peripheral DBP, DBP Z-score, 24 h HR, and all office central pressures (AoSBP, AoDBP, and AoMAP). Furthermore, LV GLS was positively correlated with end-systolic pressure (ESP) and, among biochemical variables, with serum uric acid and sodium concentrations (Figure 4).

No significant associations were found between age and any of the microRNAs analyzed (Spearman’s |R| ≤ 0.10, all *p* ≥ 0.49), nor for LV GLS (R = −0.039, *p* = 0.788).

A multivariable generalized multiple regression (GRM) analysis was performed on a group of patients with primary hypertension. The statistically significant model (R^2^ = 0.355; F = 7.88; *p* = 0.00027) identified 24 h heart rate (24 h HR), red blood cell (RBC) count, and miR-16-5p expression level (beta = 0.331, 95CI: 0.084–0.578, *p* = 0.010) as independent determinants of LV GLS (Table 4). The achieved statistical power ranged from 0.657 to 0.849, depending on the predictor.

Partial Spearman correlation analysis adjusting for age, sex, BMI Z-score, and SBP Z-score showed attenuation of the association (r = 0.24, *p* = 0.089). Further details of unadjusted and adjusted analyses are provided in Supplementary Table S2.

## 3. Discussion

Our findings showed miR-16-5p was independently positively associated with LV GLS in our cohort of hypertensive children, which persisted in multivariable regression analysis adjusting for relevant clinical covariates, pointing to a potential link with subclinical myocardial deformation. In between-group comparisons, miR-16-5p approached but did not reach conventional statistical significance. Previous studies have described miR-16-5p as a contributing factor linked to adverse cardiovascular remodeling and complications in various disease states, particularly in adult populations [15,25].

Furthermore, miR-27b-3p differed significantly between hypertensive and control groups and was negatively correlated with LV GLS in the overall cohort. This may suggest a potential protective role for this microRNA in the context of pediatric hypertension ([26] and our yet unpublished data).

Overall, our outcomes suggest that microRNAs may be components of integrated molecular signatures, in addition to isolated biomarkers. The emerging concept of theranomiRNA proposes that selected microRNAs could provide diagnostic, prognostic, and therapeutic value simultaneously in cardiovascular disease [27]. In pediatric hypertension, microRNA panels, when combined with high-level analytical approaches such as machine learning, may in the future improve risk stratification and identify early myocardial vulnerability and drug targets [28]. Given our results, miR-16-5p and miR-27b-3p should be considered in such models.

However, due to the modest effect sizes and limited sample size in this study, these observations require prudent interpretation. Validation in larger, multicenter cohorts and investigation of expanded microRNA profiles are essential prior to considering clinical implementation.

To the best of our knowledge, this is the first study in which an association between circulating miR-27b-3p expression and LV GLS in children with PH has been demonstrated. Although one previous study has reported elevated serum miR-27b levels in hypertensive adults with established left ventricular hypertrophy compared to healthy controls [16], our findings demonstrate higher circulating plasma miR-27b-3p expression in healthy children and an inverse association with myocardial deformation, as assessed by LV GLS. Interestingly, recent experimental and clinical study in chronic pressure overload states suggests that sustained upregulation of miR-27b in cardiac tissue may alleviate—by targeting the Mff/MAVS axis—myocardial injuries, which are characteristic of advanced remodeling [29]. Moreover, another recent research has demonstrated that miR-27b can counteract several harmful effects of TNF-α, such as endothelial dysfunction and mitochondrial redox imbalance; it has also been shown to reduce mitochondrial apoptosis and downregulate FOXO1 expression, which can limit the effects of the Akt/FOXO1 signaling pathway and reduce oxidative stress-driven cellular injury [26]. These ambiguous observations highlight the context- and stage-dependent functions of miR-27b in cardiovascular regulation, supporting the idea that its biological role shifts from protective in the early stages of disease to potentially detrimental in established hypertensive heart disease. This apparent discrepancy may also reflect age-dependent regulation and developmental differences in microRNA expression profiles between pediatric and adult populations. In children with early-stage primary hypertension and largely preserved cardiac structure, miR-27b may play a predominantly adaptive or compensatory role in maintaining myocardial mechanics and preserving the left ventricle’s systolic function. However, it cannot be unequivocally determined whether elevated miR-27b levels represent a compensatory adaptive response or developmental regulation. Further studies are needed to clarify the underlying mechanisms.

These findings further highlight the need for standardized and unified protocols for microRNA assessment. Differences in biological matrices and methodologies, such as the use of plasma in our study versus cardiomyocytes or serum in the above-cited reports, may substantially influence measured expression levels and their clinical interpretation. Therefore, they should be analyzed within a precise biological and clinical context.

Like miR-27b, the biological role of miR-16-5p appears to be context-dependent [30,31]. Some results of experiments on animal models indicate that reducing miR-16 could have a protective effect on the heart during conditions involving restricted blood supply and stress [32]. In mentioned study using rats to model heart damage, stopping the action of miR-16 has been found to reduce the death of heart muscle cells and lessen the heart’s damage during ischemic state [32]. When applied to clinical practice, studies have shown that circulating miR-16 is linked to a higher chance of a future myocardial infarction and negative cardiovascular outcomes in adults, suggesting that it could be a useful tool for assessing cardiovascular risk [33].

From a mechanistic perspective, miR-16 has been implicated in pathways related to cardiomyocyte injury, including the regulation of endoplasmic reticulum stress, apoptotic signaling and cellular survival cascades [34]. These pathways may contribute to impaired myocardial mechanics and vulnerability to ischemic damage.

In our cohort, we found a positive correlation between circulating miR-16-5p levels and LV GLS values and miR-16-5p expression was independent determinants of LV GLS in multivariate model. This suggests that higher microR-16-5p expression is associated with less negative (i.e., impaired) longitudinal myocardial deformation and potentially reflects early alterations in left ventricular contractile mechanics in juvenile patients with PH.

LV GLS is an STE measure that quantifies the left ventricle’s longitudinal systolic shortening and serves as a sensitive marker of subclinical ventricular dysfunction, often detecting abnormalities before changes in conventional indices, such as ejection fraction [35]. Meta-analysis by Tadic et al. demonstrates that STE can detect subclinical systolic myocardial dysfunction in hypertensive individuals, even when conventional parameters, such as left ventricular ejection fraction (LVEF), remain within the normal range [36]. Another recent meta-analysis and pediatric cohort studies have consistently demonstrated that LV GLS is less negative in hypertensive young people compared with normotensive controls [10]. This supports the concept of early, subclinical impairment of the heart’s longitudinal systolic mechanics in hypertensive children. Importantly, pediatric evidence suggests that abnormalities in myocardial strain can be detected before overt left ventricular hypertrophy develops, and that changes occur even when systolic function, as assessed by LVEF and fractional shortening (FS), is fully preserved, highlighting GLS as a sensitive marker of early hypertensive cardiac involvement [10].

Consistent with these observations, our study found significantly less negative LV GLS values in children with primary hypertension than in healthy peers, suggesting that myocardial strain analysis may provide additional value beyond conventional echocardiographic measures. Collectively, these findings underscore that LV GLS is more sensitive than LVEF for detecting subclinical myocardial dysfunction in hypertensive heart disease, reflecting early impairment of longitudinal myocardial fiber mechanics that precedes a measurable decline in LVEF. From a clinical perspective, centers with access to strain imaging may consider incorporating LV GLS into the recommended diagnostic work-up to identify subclinical myocardial involvement, even in the absence of established HMOD. This could potentially enable closer monitoring and earlier initiation or intensification of pharmacological therapy in selected patients.

In addition to impaired systolic mechanics, our hypertensive pediatric cohort demonstrated significantly lower early diastolic myocardial velocities (E′) at both the septal and lateral mitral annulus compared with controls, as well as a prolonged isovolumetric relaxation time (IVRT), indicating early diastolic dysfunction. These tissue Doppler imaging (TDI) findings are consistent with previous reports in children with primary arterial hypertension [37]. These observations support the presence of subclinical diastolic impairment in the setting of elevated blood pressure. Furthermore, meta-analytic data in pediatric hypertension demonstrate that abnormalities in diastolic function parameters are common in hypertensive children compared with normotensive controls, underscoring the substantial burden of diastolic dysfunction even in the absence of overt systolic failure [38]. The combination of reduced E′ velocities and prolonged IVRT observed in our cohort reinforces the concept that childhood hypertension is associated with early alterations in myocardial relaxation and filling dynamics. Early detection of these subclinical systolic and diastolic abnormalities using STE and TDI may provide valuable prognostic information and guide earlier therapeutic interventions to prevent progression to overt ventricular dysfunction.

Additionally, LV GLS was positively associated with blood pressure-related parameters, including DBP, AoSBP, AoDBP, and AoMAP, as well as with serum uric acid and sodium concentrations. In a recent study involving a pediatric cohort with chronic kidney disease (CKD), the association between reduced LV GLS and DBP appeared more consistent than with SBP [35]. Consistent with these observations, in our study, LV GLS was more closely associated with DBP than SBP, as significant correlations were observed with peripheral office DBP but not with peripheral office SBP. All central blood pressure parameters, including AoSBP, AoDBP and AoMAP, showed positive correlations with LV GLS in our study, which may indicate a greater role for central pressure than peripheral pressure, and for DBP than SBP, in the early changes that occur in the heart. It is noteworthy that central blood pressure parameters were not assessed in the aforementioned study. Therefore, our findings suggest that, in addition to assessing myocardial strain, evaluating central hemodynamic parameters might provide further insight into strain–blood pressure relationships during the early stages of hypertensive cardiac involvement.

Taken together, a mechanistic framework can link pediatric PH to early longitudinal myocardial dysfunction through pathways involving neurohumoral factors and oxidative stress. Pediatric PH is characterized by increased sympathetic activity and hemodynamic overdrive, which may contribute to myocardial cellular stress, including oxidative and endoplasmic reticulum stress [39,40]. Studies suggest that miR-16-5p is involved in stress response and remodeling pathways, particularly in oxidative stress signaling and the regulation of cardiomyocyte survival [34].

In this context, we can propose the following hypothesis: hypertension leads to increased adrenergic drive and oxidative stress, which, in turn, upregulate miR-16-5p, resulting in preferential impairment of longitudinal fibers and, subsequently, less negative GLS (Figure 1). While our study’s cross-sectional design prevents us from inferring causality, this framework might support the biological plausibility of using miR-16-5p as a biomarker for early myocardial deformation abnormalities in pediatric PH. Further studies with a larger cohort are needed to confirm this mechanistic framework in this context.

### Limitations and Strengths

There are some acknowledged limitations to our study that must be addressed. Firstly, the study has a narrow focus on a small set of six preselected microRNAs and is unable to perform global normalization of microRNA expression. Another limitation of the study is the absence of a formal pubertal staging assessment, as hormonal and developmental changes during puberty may influence circulating microRNA profiles and cardiac functional parameters. Although strict pre-analytical procedures were applied, hemolysis was assessed only by visual inspection and not by molecular markers (e.g., the miR-451a/miR-23a-3p ratio), which should be considered another limitation of the study. Furthermore, all patients were from a single center, which had limited ethnic and age diversity. This makes it difficult to generalize the results. Another limitation of the study was the lack of formal assessment of LV GLS reproducibility. All measurements were performed by a single experienced investigator using the same echocardiographic device, platform and software; however, intra-observer variability were not formally evaluated. Lastly, the relatively small sample size of the hypertensive cohort (*n* = 50) may have limited the statistical power of adjusted correlation analyses. Although miR-16-5p remained independently associated with LV GLS in the multivariable regression model, the partial correlation analysis showed attenuation of the association. Finally, our study has several methodological limitations related to the analytical framework of circulating miRNA quantification. Because relative real-time PCR quantification was applied and normaliza-tion relied on exogenous spike-in controls rather than validated endogenous reference miRNAs, the reported fold changes should be interpreted as relative estimates of expres-sion differences rather than absolute quantitative measurements. Consequently, the find-ings should be considered exploratory and hypothesis-generating, requiring confirmation in independent cohorts using optimized normalization strategies. However, in plasma-based circulating miRNA studies, universally stable endogenous reference miRNAs have not been consistently established yet - due to the low abundance and high biological var-iability of circulating miRNAs. However, the relatively homogeneous composition of our study cohort (untreated pharmacologically children with primary hypertension) is also a strength in a pioneering study that has never been conducted before, as it reduces biological and clinical heterogeneity and enhances internal validity. This is especially important when considering the abundance of microRNA binding sites, their multidirectional effects, and the difficulties in interpreting the results. Furthermore, our study has other key strengths. It is the first to compare microRNA expression in children and its association with LV GLS. We used a commercially available and reproducible methods to measure plasma microRNA expression, performed according to the manufacturer’s instructions. All reactions were conducted in technical triplicates, and a no-template control (NTC) was included on each plate. All analyses were conducted by a single investigator to reduce bias. Independent bodies reviewed the study protocol, and a preliminary study was conducted to validate the procedures. Additionally, we thoroughly assessed the patients’ vascular phenotype, blood pressure parameters and performed a thorough echocardiographic examination, evaluating numerous indices.

## 4. Materials and Methods

### 4.1. Study Design and Study Group

The study was designed as a prospective, cross-sectional investigation conducted according to a predefined protocol prospectively reviewed and approved by the Bioethics Committee of the Medical University of Warsaw (KB-154/2020; 14 September 2020) and externally peer-reviewed within the National Science Centre, Poland funding scheme (2021/41/N/NZ5/04194). Patients were recruited from a single Pediatric Nephrology Center from November 2021 to June 2023. The study group (SG) comprised untreated patients with PH, while the control group (CG) consisted of children without hypertension who were the same age and sex as the study participants and met the same exclusion criteria.

The inclusion criteria were as follows: a newly diagnosed case of untreated arterial hypertension, in accordance with the recommendations of the European Society of Hypertension (ESH) [41], verified using ambulatory blood pressure monitoring (ABPM) [42]. Additional eligibility criteria included a body height of at least 120 cm and written informed consent.

Patients were excluded if they had: secondary hypertension (as assessed according to the protocol described [43]), presented with significant cardiac (including heart failure), renal or hepatic disease, active inflammatory conditions, or acute infections

### 4.2. Laboratory Procedures

#### 4.2.1. General Sample Handling

All blood samples collected during the study were taken on fasting (at least 10 h without a meal) between 7–11 a.m. Blood samples were collected into 6 mL EDTA Vacutainer tubes (Becton, Dickinson and Company, Franklin Lakes, NJ, USA). They were centrifuged at 3000× *g* for 15 min at 4 °C within 10 min of blood collection, and the supernatant was centrifuged again under the same conditions to remove any trace of blood cells. In addition, hemolysis was visually inspected. Samples found to be hemolyzed were excluded from further analysis. After centrifugation, the obtained plasma was carefully transferred into two RNase- and DNase-free cryovials, each containing 1 mL of plasma, and immediately frozen at −80 °C for further analysis. Plasma samples were handled only with extended, sterile, double-filtered pipette tips free of RNase, DNase, PCR inhibitors, endotoxin, and DNA (Eppendorf ep Dualfilter T.I.P.S., Hamburg, Germany) to protect against degradation of microRNAs. There was no additional freeze–thaw cycle; all samples were used only once.

For miRNA extraction, plasma samples were thawed on ice, gently vortexed, and centrifuged to remove any remaining debris. A fixed volume of 400 µL of plasma (middle part) was used for each miRNA isolation, ensuring standardization across all study participants. At the final stage of the extraction procedure, total RNA was eluted using 100 µL of RNase/DNase-free water (molecular grade). This elution volume was consistent for all samples processed. All isolation procedures were performed using mirVana PARIS kits (Thermo Fisher Scientific, Waltham, MA, USA). RNA quantity, quality, and purity were assessed using a NanoDrop Lite spectrophotometer (Thermo Fisher Scientific, Waltham, MA, USA) (samples with an A260/280 ratio between 1.8 and 2.1 were considered acceptable for further analysis). Reverse transcription was conducted with TaqMan kits, followed by real-time PCR using TaqMan Advanced microRNA Assays (Thermo Fisher Scientific, Waltham, MA, USA) and the LightCycler 480 II (Roche Diagnostics GmbH, Mannheim, Germany). Absence of inhibition was confirmed by linear Ct shift across di-lutions (Supplementary Table S3). To reduce technical variation, reactions were carried out in triplicate, and mean cycle threshold values were used for analysis (intra-assay CV 1.0-1.9%). Replicates with SD > 0.5 Ct were excluded. Inter-assay reproducibility was assessed using identical plasma samples analyzed on independent plates (CV 2.0-3.8%). Serial di-lution experiments using pooled cDNA were performed to assess assay linearity, working range, and potential inhibition (R² values ranging from approximately 0.96 to 0.99, slopes close to the expected value for efficient amplification (~3.3) and amplification efficiencies ranged from approximately 85% to 106%). reactions were carried out in triplicate, and mean cycle threshold values were used for analysis. microRNA expression was calculated using the 2^−ΔΔCT^ method [44]. Expression values were log10-transformed before analysis to improve normality.

#### 4.2.2. Spike-In and Exogenous Normalization:

Given the absence of a validated housekeeping microRNA for normalization of microRNA content, we used *Caenorhabditis elegans* miR-39 (cel-miR-39) and miR-54 (cel-miR-54) as spike-in controls—exogenous normalizers [45]. In addition, a randomly selected miR-21-5p sample (reference sample) was included as an internal reference across all qPCR runs to ensure inter-run comparability. Its expression did not differ significantly between groups (*p* = 0.486; r = 0.07), confirming its stability and suitability as a reference gene.

After the addition of 2X Denaturing Solution to the plasma sample, the cel-miR-39 was immediately added and thoroughly mixed. As a control for the reverse transcription step, cel-miR-54 was incorporated into the RT Reaction Mix during the poly(A) tailing reaction in place of an equal volume of RNase-free water, ensuring identical concentrations across all samples.

All microRNA sequences and accession numbers (https://www.mirbase.org/; accessed on 26 January 2026) are listed in Supplementary Table S4 for reference and further analysis. The primers and probes sequences are part of the pre-formulated assay and are not publicly disclosed by the manufacturer (Thermo Fisher Scientific, Waltham, MA, USA).

Our previous manuscripts provide a detailed description of the methodology used to determine the parameters in Section 4.3 and Section 4.4 [46,47]. The kits used (according to the manufacturer’s instructions) for RNA isolation, reverse transcription, and qPCR, including catalog numbers and manufacturers, are listed in Supplementary Table S5. Reporting of real-time PCR experiments followed commonly recommended quality control princi-ples described in the MIQE guidelines (Supplementary Table S6) [48]

### 4.3. Basic Clinical and Biochemical Parameters

The following clinical variables were recorded among study participants: age, sex, duration of arterial hypertension, gestational age at birth, and birth weight. Current height, weight, and body mass index (BMI) were measured and Z-scores calculated [48]. Biochemical indices were determined using routine laboratory techniques (dry chemistry; VITROS 5600, Ortho Clinical Diagnostics, Raritan, NJ, USA) and included serum creatinine, sodium, potassium, and uric acid levels. The albumin-to-creatinine ratio was measured in a first morning urine sample. For children under 15 years old, the estimated glomerular filtration rate (eGFR) was calculated using the Schwartz equation, and for those 16 years and older, it was calculated using the CKD-EPI equation [49,50].

### 4.4. BP Measurements and Vascular Examinations

The following parameters were measured: SBP—Systolic Blood Pressure; DBP—Diastolic Blood Pressure and Z-score [51]; MAP—Mean Arterial Pressure by Omron HBP-1320 (OMRON HEALTHCARE, Co., Ltd., Kyoto, Japan); 24 h: cSBP—Central Systolic Blood Pressure; cDBP—Central Diastolic Blood Pressure; PP—Pulse Pressure; 24 h-PWV—24-Hour Pulse Wave Velocity; TVR—Total Vascular Resistance; 24 h-AIx—24-Hour Augmentation Index; CO—Cardiac Output; SV—Stroke Volume; RM—Reflection Magnitude by Mobil-O-Graph (I.E.M. Industrielle Entwicklung Medizintechnik GmbH, Stolberg, Germany); AoSBP—Aortic Systolic Blood Pressure; AoDBP—Aortic Diastolic Blood Pressure; AoMAP—Aortic Mean Arterial Pressure; AIx75HR—Augmentation Index at a Heart Rate of 75/min; Buckberg-SERV—subendocardial viability ratio; ESP—end systolic pressure by the Sphygmocor device (AtCor Medical Pty Ltd., Sydney, Australia); cIMT—common carotid artery intima–media by the Aloka Prosound Alpha 6 (Hitachi Aloka Medical, Mitaka, Japan). Both the cfPWV and the cIMT were presented as numerical values and Z-scores [23,24].

### 4.5. Echocardiography Measurements

Echocardiographic assessments were conducted with an EPIQ CVx 5.0 ultrasound machine (Philips Medical System, Andover, MA, USA) equipped with an X5-1 matrix transducer. The frequencies of the sector array used in the study ranged from 2.1 to 4.2 MHz. Two-dimensional guided M-mode, pulsed wave Doppler, tissue Doppler (TDI), and speckle tracking echocardiograms were performed in parasternal short-axis and apical views.

Global left-ventricular deformation parameters were obtained using a semiautomated method with the device mentioned above. Apical 4-, 2-, and 3-chamber views were acquired. Endocardial contours were generated automatically, after which the operator could refine the mitral annulus and apical reference points and adjust the reconstructed 3-dimensional endocardial surface when necessary. Papillary muscles were considered part of the LV cavity. The final value (LV GLS) was the average of the measurements from all three apical projections.

Left-ventricular mass (LVM) was calculated from M-mode echocardiographic measurements, including interventricular septal thickness in diastole (IVSd), left-ventricular end-diastolic diameter (LVEDd), and posterior wall thickness in diastole (LVPWd), using the Devereux equation [52]. In accordance with the European Society of Hypertension recommendations, left-ventricular mass index (LVMI) was obtained by dividing LVM (g) by height raised to the power of 2.7 (Height ^2.7^, [m]) [41]. LVMI Z-scores were subsequently derived using the reference equations proposed by Foster et al. [53].

### 4.6. Statistical Methods

The study was originally designed to evaluate circulating microRNA expression, and the sample size was calculated a priori based on expected differences in microRNA levels between groups, ensuring adequate statistical power for the primary microRNA-related outcomes (statistical power 0.80, *p* = 0.05). The present GLS analysis represents a predefined secondary analysis within this cohort.

The normality of continuous variables was assessed using the Shapiro–Wilk test. Continuous data are presented as mean ± standard deviation (SD) and interquartile range (IQR), whereas categorical variables are expressed as percentages. Depending on data distribution, comparisons were performed using Student’s *t*-test, Mann–Whitney U test, Spearman’s rank correlation, or Pearson correlation. The χ^2^ test was applied to compare categorical variables between groups. Multivariate analysis was conducted for patients in the study group using a multivariable generalized multiple regression (GRM) model and step-wise regression. Age, sex, BMI Z-score, SBP Z-score, 24 h HR, RBC count, and miR-16-5p expression level were included as clinically relevant covariates. Variables that showed significant correlations with LV GLS were included in the model, while variables with intercorrelations exceeding |r| = 0.6 were excluded to avoid collinearity. *p*-values < 0.05 were considered statistically significant. Given the sample size (n = 50), the number of predictors was restricted to reduce the risk of overfitting. Additionally, a post hoc power analysis was performed for the multivariable regression model in the hypertensive study group. Partial Spearman correlation analysis was performed to assess the association between microRNA expression and LV GLS after adjustment for age, sex, BMI Z-score, and SBP Z-score. Statistical analysis was performed using Dell Statistica 13.0 PL software (TIBCO Software Inc., Palo Alto, CA, USA).

## 5. Conclusions

MiR-16-5p was independently associated with impaired left ventricular global longitudinal strain in children with primary hypertension. This finding may support a link between molecular stress responses and early subclinical myocardial dysfunction in this patient group. MiR-27b-3p differed significantly between the hypertensive and control groups. It also correlated negatively with LV GLS in the overall cohort. This suggests that distinct microRNAs may reflect opposite aspects of hypertension-related cardiac remodeling. Both microRNAs should be considered in future theranomiRNA studies as possible biomarkers and drug targets.

LV GLS was significantly reduced in pediatric primary hypertension and may serve as a sensitive marker of early myocardial involvement whenever it is available in clinical practice. However, given the cross-sectional design and modest sample size, these findings should be interpreted cautiously and validated in larger prospective studies.

## Figures and Tables

**Table 1:** Demographic, clinical, and basic biochemical characteristics of the study group and the control group, and comparison between them.

Parameter	Study Group	Control Group	*p*
Age [years]	15.39 ± 1.90 (14.36–16.84)	15.25 ± 1.55 (13.76–16.54)	0.424
Sex [male/female]	80.0%/20.0%	70.2%/29.8%	0.243
Hypertensive Mother	32.0%	8.8%	0.003
Hypertensive Father	46.0%	28.1%	0.055
Height [cm]	173.74 ± 8.82 (168.00–180.00)	171.91 ± 10.61 (166.00–180.00)	0.368
Height Z-score	0.71 ± 1.06 (0.07–1.42)	0.61 ± 0.99 (−0.07–1.20)	0.688
Weight [kg]	79.02 ± 19.60 (64.00–85.00)	61.77 ± 11.68 (55.00–69.00)	<0.001
Weight Z-score	1.42 ± 1.10 (0.69–2.20)	0.44 ± 0.84 (0.02–1.03)	<0.001
BMI	26.17 ± 6.22 (22.21–27.94)	20.83 ± 3.46 (18.67–22.35)	<0.001
BMI Z-score	1.26 ± 1.06 (0.62–2.09)	0.16 ± 1.07 (−0.42–0.83)	<0.001
SPB [mmHg]	136.34 ± 10.42 (131.00–144.00)	118.68 ± 9.64 (113.00–125.00)	<0.001
SBP Z-score	1.76 ± 0.84 (1.26–2.30)	0.23 ± 0.82 (−0.34–0.80)	<0.001
DBP [mmHg]	82.21 ± 7.88 (76.00–88.00)	72.86 ± 6.35 (69.00–77.00)	<0.001
DBP Z-score	2.25 ± 1.10 (1.40–3.07)	0.99 ± 0.82 (0.44–1.51)	<0.001
Waist circumference [cm]	83.8 ± 14.3 (73.6–88.8)	71.1 ± 6.8 (67.5–74.5)	<0.001
Hip circumference [cm]	100.3 ± 12.0 (92.0–104.3)	91.4 ± 8.3 (86.5–96.0)	<0.001
WtH ratio	0.84 ± 0.11 (0.78–0.88)	0.78 ± 0.04 (0.75–0.81)	<0.001
Uric Acid [mg/dL]	5.81 ± 1.09 (5.10–6.30)	4.98 ± 0.88 (4.50–5.70)	<0.001
eGFR [mL/min/1.73^2^]	107.58 ± 18.00 (96.01–121.32)	106.96 ± 17.51 (92.41–116.97)	0.854
miR-16-5p expression	5.77 ± 0.43 (5.60–6.09)	5.93 ± 0.33 (5.77–6.13)	0.067
miR-27b-3p expression	5.05 ± 0.65 (4.70–5.46)	5.58 ± 0.69 (5.28–5.96)	<0.001

BMI—body mass index; SBP—systolic blood pressure; DBP—diastolic blood pressure; WtH ratio—waist-to-hip circumference ratio; eGFR—estimated glomerular filtration rate.

**Table 2:** 24 h blood pressure parameters and vascular parameters in the study group and the control group, and comparison between them.

Parameter	Study Group	Control Group	*p*
24 h SBP [mmHg]	131.60 ± 9.56 (124.00–138.00)	114.91 ± 6.92 (109.00–121.00)	<0.001
24 h DBP [mmHg]	74.06 ± 6.51 (70.00–78.00)	66.00 ± 5.14 (64.00–69.00)	<0.001
HR 24 h [1/min]	73.24 ± 9.07 (67.00–79.00)	71.21 ± 9.82 (63.00–79.00)	0.221
MAP 24 h [mmHg]	100.38 ± 7.26 (95.00–105.00)	88.44 ± 5.11 (85.00–92.00)	<0.001
PP 24 h [mmHg]	57.02 ± 7.02 (53.00–59.00)	48.42 ± 6.57 (44.00–52.00)	<0.001
AoSBP [mmHg]	114.61 ± 8.50 (110.40–118.20)	100.63 ± 7.83 (94.00–107.00)	<0.001
AoDBP [mmHg]	83.59 ± 7.94 (77.29–89.00)	74.12 ± 6.44 (70.00–78.33)	<0.001
AoMAP [mmHg]	98.18 ± 7.48 (93.17–103.20)	86.74 ± 6.73 (81.67–91.67)	<0.001
cfPWV [m/s]	5.26 ± 0.65 (4.78–5.63)	4.71 ± 0.60 (4.20–5.07)	<0.001
cfPWV Z-score	−0.20 ± 0.85 (−0.80–0.19)	−0.94 ± 0.80 (−1.44–−0.30)	<0.001
IMT mean [mm]	0.49 ± 0.05 (0.45–0.53)	0.46 ± 0.04 (0.43–0.49)	0.010
IMT Z-score	1.87 ± 1.08 (1.27–2.71)	1.41 ± 0.85 (0.67–2.00)	0.020

24 h SBP—24 h mean systolic blood pressure; 24 h DBP—24 h mean diastolic blood pressure; HR 24 h—24 h mean heart rate; MAP 24 h—24 h mean arterial pressure; PP 24 h—24 h pulse pressure; AoSBP—central (aortic) systolic blood pressure; AoDBP—central (aortic) diastolic blood pressure; AoMAP—central (aortic) mean arterial pressure; cfPWV—carotid–femoral pulse wave velocity; cfPWV Z-score—Z-score of carotid–femoral pulse wave velocity adjusted for height and sex [23]; IMT mean—mean carotid intima–media thickness. IMT Z-score—carotid intima–media thickness adjusted for height and sex [24].

**Table 3:** Echocardiography-derived parameters with extended indices of systolic cardiac function in the study and control groups, and a comparison between them.

Parameter	Study Group	Control Group	*p*
LVM [g]	145.60 ± 44.12 (110.76–165.03)	122.07 ± 32.00 (103.40–142.60)	0.008
LVMI [g/m^2.7^]	32.49 ± 8.45 (26.05–36.67)	28.00 ± 5.94 (22.68–32.30)	0.005
LVMI [g/m^2^]	74.59 ± 18.69 (62.88–87.87)	70.66 ± 14.05 (62.96–79.90)	0.342
LVMI Z-score	−0.66 ± 1.52 (−1.64–0.28)	−1.36 ± 1.47 (−2.51–−0.05)	0.014
**LV GLS [%]**	**−19.28 ± 3.02 (−21.30–−16.90)**	**−20.99 ± 3.11 (−23.00–−19.70)**	**0.002**
E-wave velocity [m/s]	90.19 ± 15.60 (79.30–100.00)	89.22 ± 13.09 (81.00–97.30)	0.776
A-wave	55.57 ± 9.67 (46.70–63.10)	53.05 ± 11.70 (45.50–58.20)	0.103
E/A	1.67 ± 0.39 (1.39–1.92)	1.72 ± 0.27 (1.53–1.93)	0.159
E’ sept [cm/s]	12.73 ± 2.34 (11.05–13.85)	14.46 ± 2.35 (13.00–15.70)	<0.001
A’ sept [cm/s]	7.44 ± 1.74 (6.31–8.59)	7.56 ± 1.75 (6.22–8.70)	0.959
E’ lat	17.25 ± 3.68 (14.50–18.90)	19.10 ± 2.95 (17.00–20.20)	0.004
A’ lat	7.52 ± 1.90 (6.20–8.81)	7.26 ± 1.78 (5.91–8.27)	0.340
E/E’ sept	7.23 ± 1.43 (6.12–7.95)	6.33 ± 1.28 (5.39–7.17)	0.001
E/E’ lat	5.34 ± 0.96 (4.58–5.95)	4.77 ± 0.93 (4.03–5.34)	<0.001
IVCT [ms]	69.64 ± 16.77 (58.00–79.00)	70.95 ± 15.38 (61.00–78.00)	0.965
IVRT [ms]	74.66 ± 13.34 (63.00–86.00)	65.71 ± 14.47 (58.00–69.00)	<0.001

LVM—left ventricular mass; LVMI—left ventricular mass index LVMI; LV GLS—left ventricular global longitudinal strain; E-wave velocity—early diastolic transmitral inflow velocity; A-wave—late diastolic transmitral inflow velocity; E/A—ratio of early to late transmitral inflow velocities; E’ sept—septal early diastolic mitral annular velocity; A’ sept—septal late diastolic mitral annular velocity; E’ lat—lateral early diastolic mitral annular velocity; A’ lat—lateral late diastolic mitral annular velocity; E/E’ sept—ratio of transmitral E-wave velocity to septal E’ velocity; E/E’ lat—ratio of transmitral E-wave velocity to lateral E’ velocity; IVCT—isovolumetric contraction time; IVRT—isovolumetric relaxation time. Bold values indicate the primary study parameter (LV GLS).

**Table 4:** Multivariate analysis of factors influencing global left ventricle strain in the group of hypertensive children (generalized multiple regression; step-wise regression).

Analyzed Parameter	B (SE)	t	*p*	95% CI for B	Standardized β	95% CI for β
Intercept (constant)	−51.7 (6.79)	−7.62	<0.001	−65.39 to −38.01	-	-
RBC [× 10^6^/µL]	2.40 (0.79)	3.05	0.0039	0.81 to 3.98	0.375	0.13–0.62
24 h HR [1/min]	0.09 (0.04)	2.41	0.0202	0.02 to 0.17	0.297	0.05–0.54
miR-16-5p expression	2.24 (0.83)	2.70	0.0098	0.57 to 3.92	0.331	0.08–0.58

B—unstandardized regression coefficient; SE—standard error; β—standardized regression coefficient; CI—confidence interval; 24 h HR—mean 24 h heart rate in ambulatory blood pressure monitoring; RBC—red blood cell count. The intercept (constant) represents the expected GLS value when all covariates are set to zero. Model derived using a generalized linear model with stepwise variable selection.

## Data Availability

Due to the preliminary nature of the study and the need for further investigation, the data presented in this study are available on request from the corresponding author.

## Supplementary Materials

The following supporting information can be downloaded at: https://www.mdpi.com/article/10.3390/ijms27062806/s1.

## Abbreviations

The following abbreviations are used in this manuscript:

PH	Primary Hypertension
LV	Left Ventricle
GLS	Global Longitudinal Strain
STE	Speckle-tracking Echocardiography
TDI	Tissue Doppler Imaging
BMI	Body mass index
SBP	Systolic Blood Pressure
DBP	Diastolic Blood Pressure
MAP	Mean Arterial Pressure
cSBP	Central Systolic Blood Pressure
cDBP	Central Diastolic Blood Pressure
PP	Pulse Pressure
24 h-PWV	24 h Pulse Wave Velocity
TVR	Total Vascular Resistance
24 h-AIx	24 h Augmentation Index
CO	Cardiac Output
SV	Stroke Volume
RM	Reflection Magnitude
AoSBP	Aortic Systolic Blood Pressure
AoDBP	Aortic Diastolic Blood Pressure
AoMAP	Aortic Mean Arterial Pressure
AIx75HR	Augmentation Index at a Heart Rate of 75/min
Buckberg-SERV	Subendocardial viability ratio
ESP	End systolic pressure
