# Supplementary material for: Circulating miR-16 as an Early Biomarker of Subclinical Myocardial Strain Impairment in Pediatric Primary Hypertension
Source: Int J Mol Sci. 2026 Mar 20;27(6):2806. doi: 10.3390/ijms27062806 (PMC13026157; doi:10.3390/ijms27062806)
Supplement: Supplementary file 1 [file ijms-27-02806-s001.zip › ijms-4145856-Supplementary Material.pdf]

Table S1. Additional clinical, biochemical, blood pressure, vascular, and echocardiographic parameters measured during the study and their comparison between the hypertensive and control groups.

Parameter	Study group (N=50)	Control group (N =57)	p
Birth weight [g]	3270 ± 532 (3100–3500)	3346 ± 579 (3200–3650)	0.147
Duration of pregnancy [weeks]	38.56 ± 2.37 (37.50–40.00)	38.75 ± 1.92 (38.00–40.00)	0.688
Duration of hypertension [months]	10.87 ± 14.65 (0.75–15.00)	NA	NA
ACR [mg/g]	10.55 ± 18.33 (2.68–7.49)	7.73 ± 7.28 (3.58–8.49)	0.266
cSBP 24h [mmHg]	114.80 ± 8.62 (109.00–120.00)	100.35 ± 5.50 (96.00–104.00)	<0.001
cDBP 24h [mmHg]	76.52 ± 6.72 (72.00–81.00)	67.75 ± 5.30 (65.00–72.00)	<0.001
Aix-t-75HR 24h	14.92 ± 5.95 (10.00–19.00)	12.21 ± 6.25 (8.00–15.00)	0.014
CO 24h	4.76 ± 0.43 (5.00–5.00)	4.51 ± 0.50 (4.00–5.00)	0.025
TVR 24h	1562.86 ± 126.49 (1471.00–1619.00)	1460.88 ± 106.16 (1376.00–1540.00)	<0.001
RM 24h	5634.00 ± 623.55 (5400–6100)	5378.95 ± 553.15 (5000–5700)	0.010
PWV 24h [m/s]	4.70 ± 0.46 (4.00–5.00)	4.04 ± 0.19 (4.00–4.00)	<0.001
SV 24h	74.70 ± 7.74 (71.00–80.00)	73.68 ± 10.33 (66.00–82.00)	0.702
Dipping SBP [%]	11.16 ± 6.06 (7.20–15.50)	10.68 ± 4.63 (7.34–13.45)	0.465
Dipping DBP [%]	14.32 ± 6.79 (9.41–19.18)	17.50 ± 6.14 (13.43–22.08)	0.014
AIx [%]	-7.06 ± 11.37 (-15.75–0.67)	-4.42 ± 10.89 (-13.50–1.00)	0.177
AIx@HR75 [%]	-6.96 ± 11.32 (-14.20–1.80)	-7.62 ± 10.22 (-13.75–-2.20)	0.968
S-wave	50.05 ± 11.92 (40.70–59.40)	45.90 ± 8.61 (41.55–51.40)	0.167
D-wave	64.89 ± 11.01 (57.15–72.85)	64.21 ± 9.79 (56.70–69.90)	0.758

**S/D**	**0.78 ± 0.21 (0.69–0.81)**	**0.74 ± 0.18 (0.67–0.77)**	**0.202**
**AC-R**	**39.33 ± 9.88 (32.10–47.60)**	**39.11 ± 8.26 (36.00–44.50)**	**0.907**
**AC-CD**	**-30.46 ± 8.27 (-36.90–-24.70)**	**-30.62 ± 6.61 (-34.70–-27.20)**	**0.518**
**AC-CT**	**-8.87 ± 5.27 (-11.20–-6.00)**	**-8.49 ± 4.71 (-12.50–-4.50)**	**0.730**
**Ed-R**	**42.81 ± 12.71 (33.90–53.90)**	**41.75 ± 10.18 (37.60–49.00)**	**0.846**
**ED-CD**	**-33.05 ± 9.27 (-41.70–-25.90)**	**-32.78 ± 7.50 (-37.70–-29.00)**	**0.820**
**ED-CT**	**-9.77 ± 6.37 (-12.40–-5.60)**	**-8.96 ± 5.62 (-13.40–-4.70)**	**0.704**

ACR – urinary albumin-to-creatinine ratio; cSBP 24h – 24-hour central systolic blood pressure; cDBP 24h – 24-hour central diastolic blood pressure; AIx-t-75HR 24h – 24-hour augmentation index normalized to a heart rate of 75 beats per minute; CO 24h – 24-hour cardiac output; TVR 24h – 24-hour total vascular resistance; RM 24h – 24-hour reflection magnitude; PWV 24h – 24-hour pulse wave velocity; SV 24h – 24-hour stroke volume; Dipping SBP – nocturnal systolic blood pressure dipping percentage; Dipping DBP – nocturnal diastolic blood pressure dipping percentage; AIx – augmentation index; AIx@HR75 – augmentation index normalized to a heart rate of 75 beats per minute; S-wave – systolic pulmonary venous flow velocity; D-wave – diastolic pulmonary venous flow velocity; S/D – ratio of systolic to diastolic pulmonary venous flow velocities; AC-R – acceleration rate; AC-CD – acceleration time (cardiac cycle–derived); AC-CT – acceleration time (carotid tonometry–derived); Ed-R – ejection duration rate; ED-CD – ejection duration (cardiac cycle–derived); ED-CT – ejection duration (carotid tonometry–derived).

Table S2. Unadjusted and adjusted analyses of the association between microRNA-16 and LV GLS in the hypertensive group.

Analysis	Estimate	p-value
Spearman correlation (unadjusted)	r = 0.305	0.031
Partial Spearman correlation (adjusted for age, sex, BMI Z-score, SBP Z-score)	r = 0.243	0.089
Multivariable GRM (adjusted for age, sex, BMI Z-score, SBP Z-score, 24h HR, RBC)	β = 0.331	0.0098

BMI - body mass index, Z-score - , SBP - systolic blood pressure (office), GRM - generalized regression model, 24h - 24-hour, HR - heart rate, RBC - red blood cells. β denotes the standardized regression coefficient.

Table S3. Analytical validation of real-time PCR assays.
Summary of amplification performance, intra-assay precision, inter-assay reproducibility, and dilution linearity parameters for the RT-qPCR assays used in the study.

Assay	Intra-assay CV (%)	Inter-assay CV (%)	Slope	Efficiency (%)	R^2
cel-miR-39	1.1	2.6	3.27	102%	0.999
cel-miR-54	1.3	2.8	3.19	106%	0.988
miR-16	1.2	2.4	3.22	104%	0.997
miR-21	1.3	2.8	3.21	105%	0.998
miR-27a	1.3	2.1	3.41	96%	0.971
miR-27b	1.9	3.8	3.75	85%	0.999
miR-145	1.0	2.0	3.18	106%	0.969
miR-133a	1.3	2.7	3.27	102%	0.999

Table S4. Studied microRNA sequences with the corresponding accession numbers from miRbase.

microRNA	miRBase ID	Sequence
cel-microRNA-39	MIMAT0000010	UCACCGGGUGUAAAUCAGCUUG
cel-microRNA-54	MIMAT0000025	UACCCGUAAUCUUCAUAAUCCGAG
microRNA-16-5p	MIMAT0000069	UAGCAGCACGUAAAUAUUGGCG
microRNA-21-5p	MIMAT0000076	UAGCUUAUCAGACUGAUGUUGA
microRNA-27a-3p	MIMAT0000084	UUCACAGUGGCUAAGUUCCGC
microRNA-27b-3p	MIMAT0000419	UUCACAGUGGCUAAGUUCUGC

microRNA-133a-3p	MIMAT0000427	UUUGGUCCCCUUCAACCAGCUG
microRNA-145-5p	MIMAT0000437	GUCCAGUUUUCCCAGGAAUCCCU

Table S5. Commercial kits and reagents used for microRNA analysis

Application	Product Name	Catalog Number	Manufacturer
RNA isolation	miRVana™ PARIS™ Kit	AM1556	Thermo Fisher Scientific, Waltham, MA USA
cDNA synthesis	TaqMan™ MicroRNA Reverse Transcription Kit	A28007	Thermo Fisher Scientific, Waltham, MA USA
miRNA-specific assays (stem-loop RT-qPCR)	TaqMan™ MicroRNA Assays	A25576	Thermo Fisher Scientific, Waltham, MA USA
qPCR Master Mix	TaqMan™ Universal PCR Master Mix II, no UNG	4444557	Thermo Fisher Scientific, Waltham, MA USA

Table S6. MIQE guideline compliance summary for the real-time PCR experiments.

MIQE Category	Item	Information provided in this study
Sample	Biological material	Human plasma samples obtained from pediatric patients with primary hypertension and control subjects
Sample handling	Storage conditions	Plasma samples were stored at −80°C prior to RNA extraction
RNA extraction	Extraction method	miRNA extracted using a commercial extraction kit (specified in the Methods section)
RNA quality	RNA integrity assessment	Not applicable for plasma miRNA due to low RNA abundance; extraction efficiency monitored using exogenous spike-in controls
Reverse transcription	RT assay	TaqMan Advanced miRNA cDNA Synthesis Kit
qPCR assay	Detection chemistry	TaqMan Advanced miRNA assays
qPCR platform	Instrument	LightCycler 480 (Roche Diagnostics)
Replicates	Technical replicates	All reactions were performed in technical triplicates

MIQE Category	Item	Information provided in this study
Replicate QC	Variability threshold	Replicate sets with a standard deviation (SD) > 0.5 Ct were excluded according to predefined quality control criteria
Amplification efficiency	Efficiency estimation	Estimated from serial dilution experiments using pooled cDNA derived from 10 plasma samples
Dynamic range	Dilution series	Five ten-fold dilution steps were analysed
Intra-assay precision	Technical reproducibility	Mean Ct coefficient of variation (CV) approximately 1–2%
Inter-assay precision	Plate-to-plate reproducibility	Mean Ct CV approximately 2–3%
PCR inhibition	Inhibition assessment	Absence of significant PCR inhibition supported by linear Ct shift across serial dilutions
Controls	Negative control	No-template controls (NTC) containing nuclease-free water were included on each RT-qPCR plate
Normalisation	Strategy	Exogenous spike-in controls (cel-miR-39 and cel-miR-54) used to control extraction and reverse transcription variability
Quantification	Expression analysis	Relative quantification performed using the 2−ΔΔCt method

Analytical validation of RT-qPCR assays

Analytical validation of the RT-qPCR assays was performed to evaluate amplification performance, technical precision, and potential sources of variability.

To assess amplification characteristics, a serial dilution experiment was performed using pooled cDNA derived from 10 randomly selected plasma samples. Five tenfold dilution steps were analysed to evaluate amplification linearity and dynamic range. The relationship between Ct values and template dilution was assessed by linear regression, and amplification efficiency was estimated from the slope of the regression line.

Technical reproducibility was evaluated at two levels. **Intra-assay precision** was assessed using technical replicates within individual real-time PCR plates. The variability between replicates was quantified using the standard deviation (SD) and coefficient of variation (CV) of Ct values. **Inter-assay precision** was evaluated using identical plasma samples analysed on two independent real-time PCR runs. Each

sample was analysed in triplicate, and the coefficient of variation (CV) of Ct values was calculated.

Quality control criteria were applied to replicate measurements to minimise technical variability. Replicate sets with a standard deviation (SD) greater than 0.5 Ct were excluded according to predefined quality control criteria. The results of these analytical validation analyses are summarised in **Table S1**.

Additionally, samples from hypertensive patients and controls were analysed across the same real-time PCR plates to minimise potential batch effects and systematic differences between runs.
